# It's gall relative: metabolic profiling of two morphologically distinct oak leaf galls induced by cynipid wasps

**DOI:** 10.1093/plphys/kiae032

**Published:** 2024-01-22

**Authors:** Jiawen Chen

**Affiliations:** Assistant Features Editor, Plant Physiology, American Society of Plant Biologists; Division of Crop Biotechnics, Department of Biosystems, KU Leuven, 3001 Leuven, Belgium

Plant galls are outgrowths on plant tissue induced by another organism. Galls benefit the inducer, which can come from a wide range of kingdoms, including bacteria, fungi, nematodes, plants, mites, and insects (Gatjens-Boniche 2019, Harris and Pitzschke 2020). Gall formation differs from regular plant development. They display a variety of forms depending on their inducer and the species and tissue of the host, and they are referred to as an extended phenotype of the inducer. Galls can be part of a mutualistic relationship with the host, such as in plant root nodules, but they are often parasitic (Harris and Pitzschke 2020). These biotic interactions present a unique feat of bioengineering by the inducer, requiring genetic and structural reprogramming of the host.

Galls induced by microbes are well studied compared to those of insects. They tend to be relatively structurally simple, and they are also called tumors (Gatjens-Boniche 2019). A famous example is the crown gall induced by *Agrobacterium tumefaciens* (Nester 2015), which demonstrates how bioengineering in the natural world can be co-opted for bioengineering in the laboratory; *Agrobacterium* is now used extensively for plant genetic transformation. In contrast, galls induced by insects can be structurally complicated and diverse, especially those induced by cynipid wasps (Hymenoptera order, Cynipidae family) on oaks, also called oak gall wasps (Stone and Cook 1998). These wasps lay their eggs on oak tissues, and this action induces the formation of a gall structure around the eggs, to provide nutrition and protection for the larvae. Depending on the species and behavior of the wasp, one or multiple larvae develop within a single gall. Despite the large diversity in oak gall morphologies, the general structure consists of an inner larval chamber surrounded by a thin sclerenchyma cell layer and an outer gall consisting of an airspace and multiple cell layers (Stone and Cook 1998) (Fig. A). The larval chamber contains nutritive tissue that feeds the larva. It is structurally similar among cynipid galls, and transcriptomics from one type of cynipid gall on red oak shows that the larval chamber is more closely associated with the insect, whereas the outer gall is more similar to a modified plant leaf (Martinson et al. 2022), and this is where gall morphological diversity occurs (Stone and Cook 1998).

**Figure.**
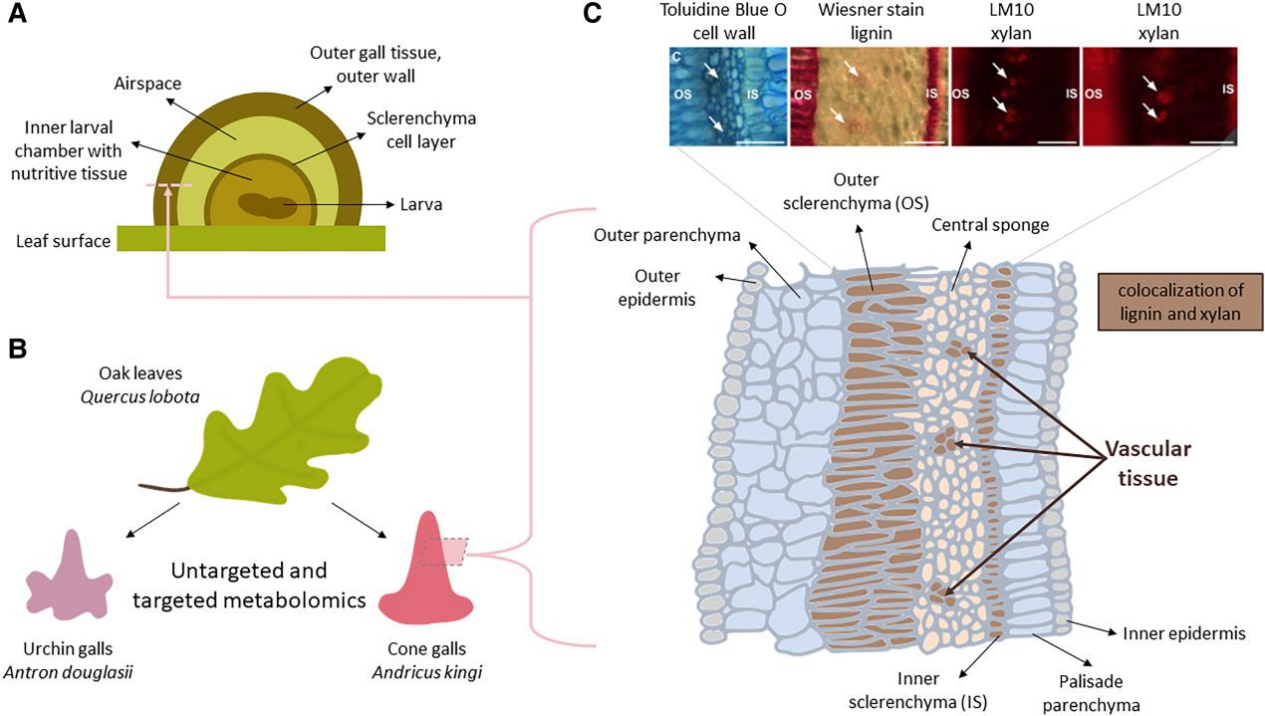
Oak galls have metabolically and structurally distinct characteristics compared to uninfected leaf tissue. **A)** Schematic of the general structure of a cynipid wasp–induced oak leaf gall, as a longitudinal section. The pink dashed line indicates the outer gall tissue layer that the transverse section from **C)** refers to. **B)** Summary of the metabolomics experiment setup from this study, where tissue from urchin galls and cone galls induced by different species of cynipid wasp were compared to each other and oak leaf tissue without galls. A dashed rectangle indicates where sections from cone galls were taken for histological staining. **C)** Schematic of a transverse section from the outer gall tissue of a cone gall, based on Supplemental Figure 10A from Markel et al. (2023), showing 7 distinct cell layers that were visualized by histological staining. The layers stained for lignin and xylan are colored brown, which consist of the inner and outer sclerenchyma and bundles consistent with vascular tissue within the central sponge layer. Above the schematic, Figure 4C from Markel et al. (2023) shows the microscopy images of the staining of transverse sections with Toluidine Blue O, Wiesner stain, and Alexa-fluor 647 secondary antibody conjugated to LM10 primary antibody. Scale bar = 50 *µ*m.

Although many different oak gall structures have been described, we still do not understand the molecular mechanism behind these unique cross-kingdom interactions. One hypothesis is that changes in hormone gradients play an important role in insect–gall interactions (Tooker and Helms 2014), and effector proteins were shown to be important for galling aphids (Korgaonkar et al. 2021). However, these are likely not to be the sole cause of the drastic changes that happen in plant galls. For cynipid wasps, previous studies have focused on the transcriptomic changes of galls, for *Biorhiza pallida* on the oak *Quercus robur* throughout different stages of gall development (Hearn et al. 2019) and for *Dryocosmus quercuspalustris* on *Quercus rubra* in different layers of gall tissue (Martinson et al. 2022). The many different ways in which cynipid wasps manipulate their plant host tissue likely involve a series of coordinated processes, such as protein regulation of plant developmental processes and enzyme activities that alter plant physiology (Hearn et al. 2019), including the breakdown and remodeling of cell walls. This makes the cynipid wasp gall a key component to study, not only in the context of plant protection against pathogens but also for our fundamental understanding of biotic interactions and plant developmental reprogramming.

In this issue of *Plant Physiology*, Markel et al. (2023) expanded on the available transcriptomic resources for oak galls by exploring the biochemical consequences of gene expression changes using metabolomics. They studied two species of cynipid wasp galls on *Quercus lobota* oak leaves, *Antron douglasii* which produces urchin galls and *Andricus kingi* which produces cone galls (Fig. B). Strikingly, histological staining of the cone galls revealed cell layer–specific alteration of lignin and polysaccharide composition, indicating de novo–formed vascular bundles.

The authors compared the two morphologically distinct structures of these galls. Laser ablation tomography (LAT) allowed for detailed 3D characterization, confirming the common gall structures as described in the literature (Fig. A). Untargeted and targeted metabolomics across different stages of oak gall development showed distinct profiles of the two species of galls compared with each other and with uninfected leaf tissue. The authors took a broad perspective to categorize patterns of metabolite changes using Natural Product Classifier and network analysis, showing, for instance, that both galls had an increase in flavonoids and a decrease in auxin and fatty acids. There were also gall type–specific effects, such as increased abscisic acid concentration in urchin galls and a reduction of trehalose in cone galls, which could both be involved in signaling pathways.

To provide not only a chemical but also a spatial view of metabolic changes in galls, the authors performed histochemical staining in the cone galls, which were more abundant than the urchin galls during sampling. Wiesner reagent (phloroglucinol + HCl, a lignin stain) revealed a tight spatial regulation of lignin deposition in the outer gall tissue, located at sclerenchyma layers and within concentrated areas in the central sponge layer (Fig. C). The composition of the lignin polymers was more reminiscent of vasculature than of fiber, pointing to de novo generation of vasculature in the central sponge tissue. This was supported by the colocalization of xylan, which is an abundant polysaccharide in vascular tissue (Fig. C).

This study adds a rich metabolomic resource to the exploration of oak gall induction by cynipid wasps, a system where there is a lot of knowledge to gain because of the complexity and diversity of the gall structures. Markel et al. (2023) present metabolic similarities and differences in two species of cynipid wasp galls, emphasizing the diversity we see within the order of cynipids. This means that findings on specific cynipid wasp–oak systems are not necessarily generalizable, such as those of previous transcriptomic studies (Hearn et al. 2019, Martinson et al. 2022), which have both consistent and inconsistent conclusions when compared with the metabolomic results of the current study. The discovery of spatially distinct de novo vascularization in cone galls further highlights the diversity we see among cynipid wasp galls, as a study in *Diplolepis nodulosa* stem galls on *Rosa blanda* specifically characterized galls supported by the existing stem vasculature rather than de novo vasculature (Brooks and Shorthouse 1998). The resources created in this study will enable further exploration of the mechanisms behind neovascularization. Questions that remain include how cell wall degradation and reprogramming is coordinated, and whether this is downstream of hormonal changes or signaling by other metabolites. As the past has shown, a better understanding of all these processes could even lead to new avenues of plant bioengineering.
